# A Novel Blood‐Based Colorectal Cancer Diagnostic Technology Using Electrical Detection of Colon Cancer Secreted Protein‐2

**DOI:** 10.1002/advs.201802115

**Published:** 2019-04-16

**Authors:** Minhong Jeun, Hyo Jeong Lee, Sungwook Park, Eun‐ju Do, Jaewon Choi, You‐Na Sung, Seung‐Mo Hong, Sang‐Yeob Kim, Dong‐Hee Kim, Ja Young Kang, Hye‐Nam Son, Jinmyoung Joo, Eun Mi Song, Sung Wook Hwang, Sang Hyoung Park, Dong‐Hoon Yang, Byong Duk Ye, Jeong‐Sik Byeon, Jaewon Choe, Suk‐Kyun Yang, Helen Moinova, Sanford D. Markowitz, Kwan Hyi Lee, Seung‐Jae Myung

**Affiliations:** ^1^ Center for Biomaterials Biomedical Research Institute Korea Institute of Science and Technology (KIST) 5 Hwarangno 14‐gil Seongbuk‐gu Seoul 02792 Republic of Korea; ^2^ Health Screening & Promotion Center Asan Medical Center 88 Olympic‐ro 43‐gil Songpa‐gu Seoul 05505 Republic of Korea; ^3^ Division of Bio‐Medical Science & Technology KIST School – Korea University of Science and Technology (UST) 5 Hwarangno 14‐gil Seongbuk‐gu Seoul 02792 Republic of Korea; ^4^ Asan Institute for Life Sciences Asan Medical Center 88 Olympic‐ro 43‐gil Songpa‐gu Seoul 05505 Republic of Korea; ^5^ Department of Pathology Asan Medical Center University of Ulsan College of Medicine 88 Olympic‐ro 43‐gil Songpa‐gu Seoul 05505 Republic of Korea; ^6^ Department of Convergence Medicine University of Ulsan College of Medicine 88 Olympic‐ro 43‐gil Songpa‐gu Seoul 05505 Republic of Korea; ^7^ Department of Gastroenterology Asan Medical Center University of Ulsan College of Medicine 88 Olympic‐ro 43‐gil Songpa‐gu Seoul 05505 Republic of Korea; ^8^ Department of Medicine and Case Comprehensive Cancer Center Case Western Reserve University 10900 Euclid Ave Cleveland OH USA; ^9^ University Hospitals Seidman Cancer Center 10900 Euclid Ave Cleveland OH USA

**Keywords:** colorectal adenomas, colorectal cancer, colorectal cancer screening, electric‐field effect colorectal sensor, tumor markers

## Abstract

Colorectal cancer (CRC) is the second‐leading cause of cancer‐related mortality worldwide, which may be effectively reduced by early screening. Colon cancer secreted protein‐2 (CCSP‐2) is a promising blood marker for CRC. An electric‐field effect colorectal sensor (E‐FECS), an ion‐sensitive field‐effect transistor under dual gate operation with nanostructure is developed, to quantify CCSP‐2 directly from patient blood samples. The sensing performance of the E‐FECS is verified in 7 controls and 7 CRC samples, and it is clinically validated on 30 controls, 30 advanced adenomas, and 81 CRC cases. The concentration of CCSP‐2 is significantly higher in plasma samples from CRC and advanced adenoma compared with controls (both *P* < 0.001). Sensitivity and specificity for CRC versus controls are 44.4% and 86.7%, respectively (AUC of 0.67), and 43.3% and 86.7%, respectively, for advanced adenomas (AUC of 0.67). CCSP‐2 detects a greater number of CRC cases than carcinoembryonic antigen does (45.6% vs 24.1%), and the combination of the two markers detects an even greater number of cases (53.2%). The E‐FECS system successfully detects CCSP‐2 in a wide range of samples including early stage cancers and advanced adenoma. CCSP‐2 has potential for use as a blood‐based biomarker for CRC.

## 1 Introduction

Colorectal cancer (CRC) is one of the most commonly diagnosed cancers in the world, and is also the second‐leading cause of cancer‐related deaths worldwide.^1^, ^2^ CRC progression and death can be reduced through proper screening methods that can detect precancerous lesions and early cancer.^3^, ^4^ Current CRC screening guidelines recommend either colonoscopy or fecal immunochemical test (FIT) as the first‐tier option.^5^, ^6^ Colonoscopy is highly sensitive in the detection of CRC and adenomas, and it also enables direct removal of adenomas during a single session. However, colonoscopy involves high costs, significant resources, discomfort from bowel preparation, and a higher risk of complications such as perforation and bleeding.^5^, ^7^ The FIT is a noninvasive test that is easy to perform and inexpensive, but it does not have sufficient sensitivity for adenoma detection.^8^ A highly sensitive FIT‐fecal DNA test has also been developed, but its high cost limits its use.^5^, ^9^ Thus, a novel noninvasive and accurate test for early detection of CRC and adenomas is needed.

Detecting blood biomarkers is a promising noninvasive method for CRC screening.^10^, ^11^ Although several blood biomarkers for CRC have been proposed, only a few of them are available for clinical use.^12^, ^13^, ^14^, ^15^, ^16^, ^17^ Among the candidate biomarkers, colon cancer secreted protein‐2 (CCSP‐2) is a potentially promising blood biomarker.^18^ CCSP‐2 mRNA expression was shown to be markedly upregulated in CRCs, with colon cancer tumors showing a mean of 78‐fold higher expression compared with matched normal mucosa. Moreover, CCSP‐2 protein secreted from tumor xenograft was successfully detected in the plasma of the xenografted mouse.^18^ Based on these findings, our group recently reported a method for fluorescent imaging of CCSP‐2 that enables more sensitive and specific detection of CRC during colonoscopy,^19^ thereby bolstering the potential use of CCSP‐2 as a blood‐based biomarker for CRC diagnosis. However, the expression patterns of CCSP‐2 in CRC and adenoma tissues have not been fully evaluated, except in one study with small sample size.^19^ Moreover, the detection of CCSP‐2 in human blood has never been reported.

**Scheme 1:**
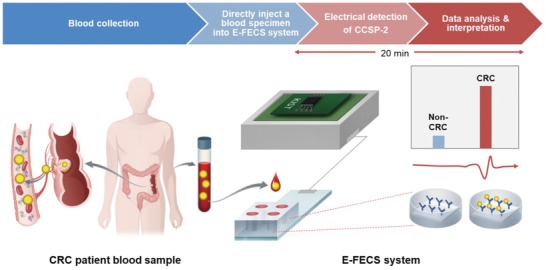
Four steps procedure for the electrical detection of CCSP‐2 biomarkers from a blood sample using the E‐FECS system. E‐FECS, electric‐field effect colorectal sensor; CCSP‐2, colon cancer secreted protein‐2; CRC, colorectal cancer.

We thus aimed to develop a safer, simpler, accurate, noninvasive system for detecting CCSP‐2 directly from human blood samples for use in early diagnosis of CRC (**Scheme** **1**). Accordingly, we developed a highly sensitive electrical CCSP‐2 detection system that consists of a disposable multiwell gate (DMWG), an integrated measurement box, and an electric‐field effect colorectal sensor (E‐FECS) chip. We optimized the system to be used in the blood environment with high reliability and sensitivity. The E‐FECS system successfully detected CCSP‐2 in plasma samples from CRC patients. Moreover, the E‐FECS system showed high sensitivity for the detection of adenoma as well. Our results demonstrate that the electrical CCSP‐2 sensing system has the potential for use as a competent blood‐based diagnostic tool for CRC.

## 2 Results

### 2.1 Electrical CCSP‐2 Detection System and Characterization

**Figure 1:**
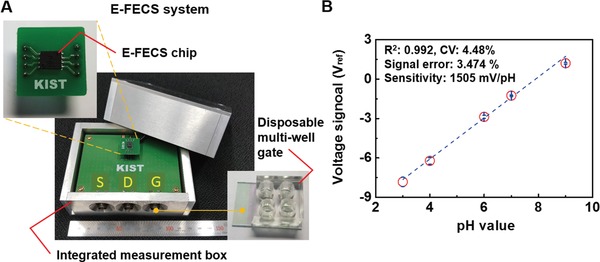
A) Photo images of the E‐FECS system: 1) an integrated measurement box which served dual purpose as a thin film transistor‐chipset input region and for transduction and amplification of detection signals, 2) a disposable multiwell gate for signal recognition. B) pH response characteristics of E‐FECS system (sensitivity: 1505 mV/pH, error range: ±3.474%, linearity: *R* ^2^ = 0.992, and CV value: 4.48%).

The electrical CCSP‐2 detection system consisted of an E‐FECS chip, a DMWG, and an integrated measurement box (**Figure** **1**A). To evaluate the system's sensing performance, we investigated back gate sensing voltage shift (Δ*V*_BG_) according to the variation of pH and compared it with the results from our previous study,^20^, ^21^, ^22^ in which we developed a dual‐gate thin film transistor (DTFT) biosensor to detect prostate cancer biomarkers including ANXA3 in urine.^20^ The current E‐FECS chip was fabricated based on the previous DTFT biosensor and the DMWG was modified for optimal operation in blood.^23^ The E‐FECS chip was sealed with an epoxy layer, which protects the degradation of the device for improving stability and reliability in blood samples. The E‐FECS chip with the DMWG showed a sensitivity of 1505 mV/pH with low error range (±3.474%), high linearity (*R* ^2^ = 0.992), and low CV value (4.48%) (Figure 1B). These numerical values are comparable with those of our previous DTFT biosensor.

### 2.2 Modification of the DMWG Surface for Optimal Operation in Blood

**Figure 2:**
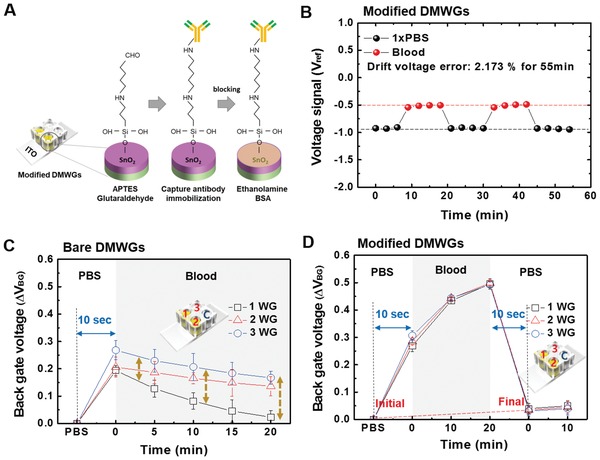
A) A chemical process to modify the surface of disposable multiwell gates (DMWGs) to improve the biomarker sensing performance in a blood environment. B) Hysteretic characteristics of electrical signals of the modified DMWGs measured in undiluted 1× PBS and blood environment for 55 min. The modified DMWGs showed high hysteretic behaviors in both 1× PBS and blood. C,D) show comparison results of electrical stability between C) bare DMWGs and D) modified DMWGs measured in both 1× PBS and blood depending on time flow. For the test, Δ*V*_BG_ were measured in each well of bare DMWGs and modified DMWGs. All Δ*V*_BG_ measured in the bare DMWGs was widely separated that means the bare DMWGs have an unstable surface. In the case of modified DMWGs, all the Δ*V*_BG_ were shown in same position.

The surface of DMWG was modified to allow stable operation in the blood environment (**Figure** **2**A). We investigated the real‐time hysteresis characteristic of the voltage signal between 1× PBS and blood to evaluate the stability and reliability of the E‐FECS chip with modified DMWG. The modified DMWG showed highly reliable response characteristics as well as good stability with 2.713% error range for 55 min (Figure 2B). Figure 2C,D show the shift tendencies of Δ*V*_BG_ of the DMWGs connected with E‐FFES chip before and after surface modification, respectively. We prepared three wells in the DMWGs with and without surface modification (bare and modified DMWGs, Figure 2C,D, respectively), and compared their shifts characteristic of the Δ*V*_BG_ in blood. Figure 2C shows the tendencies of the Δ*V*_BG_ of the bare DMWGs. All tests were initiated in PBS solution and changed to blood after 10 sec. Bare DMWGs showed marked fluctuations in Δ*V*_BG_ values, with its standard deviation (SD) increasing from 39.85 mV (0 min) to 75.85 mV (20 min) as time passed (Table S1, Supporting Information). The modified DMWGs demonstrated significantly lower Δ*V*_BG_ SD values, as the initial SD values were 18.80 mV (0 min) and decreased to 2.99 mV (20 min) as time passed. The average Δ*V*_BG_ between initial and final PBS (time interval: 20 min) solutions was 34.9 mV, was deemed as the limit‐of‐detection (LOD) voltage for distinguishing between positive and negative in the CCSP‐2 detection test.

### 2.3 Immunohistochemical Expression of CCSP‐2 in CRC Tissues

**Figure 3:**
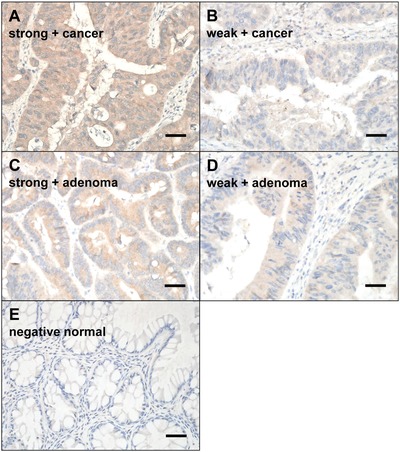
Representative immunostaining results of CCSP‐2 in human colorectal tissues. CCSP‐2 was homogenously expressed in all colorectal cancer and adenoma tissue, whereas CCSP‐2 was not detected in normal colorectal tissue. A) Strong positive in cancer, B) weak positive in cancer, C) strong positive in adenoma, D) weak positive in adenoma, and E) no signal in normal tissue. The expression profiles of CCSP‐2 were scored according to staining intensity (0, negative; 1+, weak positive; 2+, strong positive). Original magnification, ×200; Scale bar, 100 µm.

The expression of CCSP‐2 in CRC and adenoma cells was confirmed by immunohistochemistry (IHC) using 69 CRC tissues, 10 adenoma tissues, and 79 paired normal tissues (**Figure** **3**A–E) (**Table** **1**B). CCSP‐2 was homogenously expressed in all CRC and adenoma tissues, and not in normal colorectal tissues. Among the CRC tissues, 30 (43.5%) were strong positive and 39 (56.5%) were weak positive. As for the adenoma tissues, 4 (40.0%) were strong positive and 6 (60.0%) were weak positive. In the CRC tissues, both the weak‐positive and strong‐positive groups showed similar clinicopathologic characteristics, except that all poorly differentiated carcinomas were weak positive (Table S2, Supporting Information).

**Table 1:** Clinicopathologic characteristics of controls and colorectal tumor patients (BRC, Bio‐Resource Center)

A) E‐FECS verification set					
Variables				Blood	
				Control (*n* = 7)	Cancer (*n* = 7)
Age, years, median (range)			65 (32–79)		58 (49–72)
Sex, male, no. [%]			3 (42.9%)		3 (42.9%)
Location, no. [%]a)					
Proximal			–		1 (14.3%)
Distal			–		6 (85.7%)
Stage, no. [%]					
II			–		5 (71.4%)
III			–		2 (28.6%)
Differentiation, no. [%]					
Well			–		1 (14.3%)
Moderate			–		6 (85.7%)
Poor			–		0 (0.0%)
CEA, ng mL^‐1^, median (range)			–		2.1 (1.3–9.2)
B) Clinical validation set					
Variables	BRC samples (Tissue and blood)		Prospectively collected samples (Blood)		
	Adenoma (*n* = 10)	Cancer (*n* = 70)b)	Control (*n* = 30)	Adenoma (*n* = 20)	Cancer (*n* = 11)
Age, years, median (range)	59 (42–70)	59 (30–80)	56 (38–70)	62 (33–78)	62 (51–75)
Sex, male, no. [%]	5 (50.0%)	38 (54.3%)	17 (56.7%)	12 (60.0%)	6 (54.5%)
Location, no. [%]^a)^					
Proximal	4 (40.0%)	33 (47.1%)	–	11 (55.0%)	3 (27.3%)
Distal	6 (60.0%)	37 (52.9%)	–	19 (45.0%)	8 (72.7%)
Stage, no. [%]					
0	–	5 (7.1%)	–	–	5 (45.5%)
I	–	15 (21.4%)	–	–	1 (9.1%)
II	–	15 (21.4%)	–	–	2 (18.2%)
III	–	15 (21.4%)	–	–	1 (9.1%)
IV	–	20 (28.6%)	–	–	2 (18.2%)
Differentiation, no. [%]					
Well	–	10 (14.3%)	–	–	4 (36.4%)
Moderate	–	56 (80.0%)	–	–	63 (77.8%)
Poor	–	4 (5.7%)	–	–	7 (63.6%)
CEA, ng mL ^−1^, median (range)	1.3 (0.8–2.4)	1.9 (0.3–1380.0)	–	–	1.4 (0.9–390.0)

^a)^ Proximal colon includes cecum, ascending colon, and transverse colon; Distal colon includes descending colon, sigmoid colon, and rectum

^b)^ One colorectal cancer specimen without adequate cancer tissue was excluded from the tissue immunostaining analysis.

### 2.4 Comparing CCSP‐2 Detection Performance of E‐FECS and ELISA

**Figure 4:**
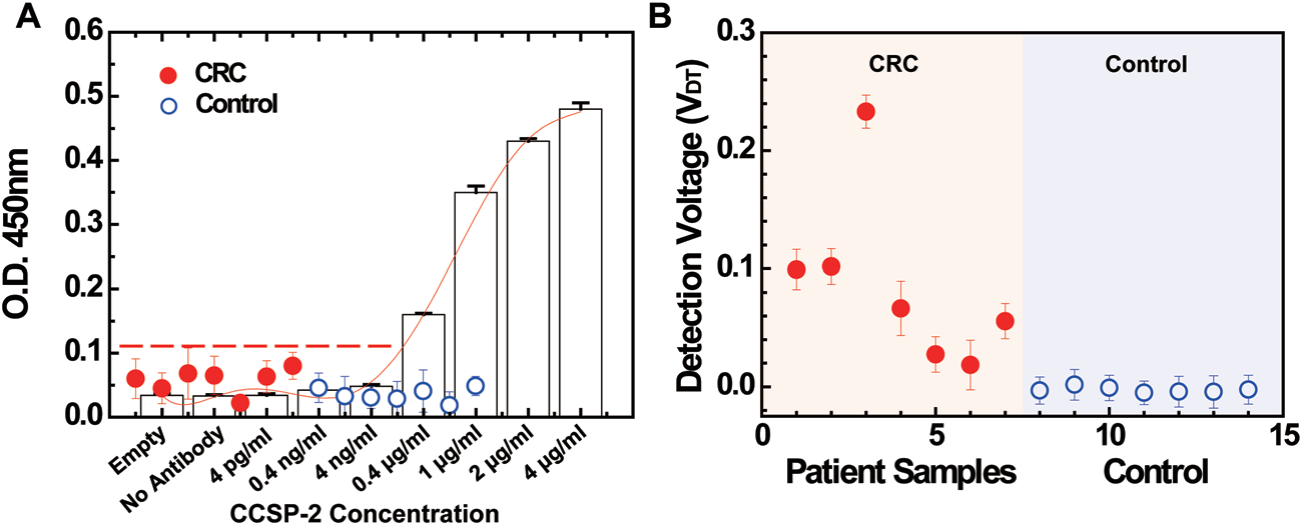
CCSP‐2 sensing performance of E‐FECS was validated in real colorectal cancer (CRC) and control samples and the results were compared with those of an ELISA. A) ELISA cannot recognize CCSP‐2 biomarkers in all the CRC samples but B) in the case of E‐FECS, successfully detected CCSP‐2 in all the CRC samples. All the samples were tested in triplicate.

CCSP‐2 detection test of the E‐FECS system was initially conducted using a verification set of human blood specimens (7 CRC and 7 normal blood samples, Table 1A), and compared its performance with those of enzyme‐linked immunosorbent assay (ELISA) results. We first conducted CCSP‐2 detection using ELISA to confirm the specificity and affinity of the anti‐CCSP‐2 antibody to the CCSP‐2 protein, and to validate the detection performance of ELISA. **Figure** **4**A shows the optical density values of the ELISA results across the range of input CCSP‐2 concentrations, demonstrating clear detection of CCSP‐2 protein but with a lower LOD (0.4 µg mL^−1^), thus lacking optimal sensitivity for detecting CCSP‐2 protein from the blood of either CRC (red dots) or control (open blue dots) individuals. In contrast, the E‐FECS system successfully detected CCSP‐2 in 5 out of 7 CRC blood samples, and none in any of the 7 control blood samples (Figure 4B).

### 2.5 Comparing CCSP‐2 Detection by the E‐FECS System in PBS, Plasma, and Serum

**Figure 5:**
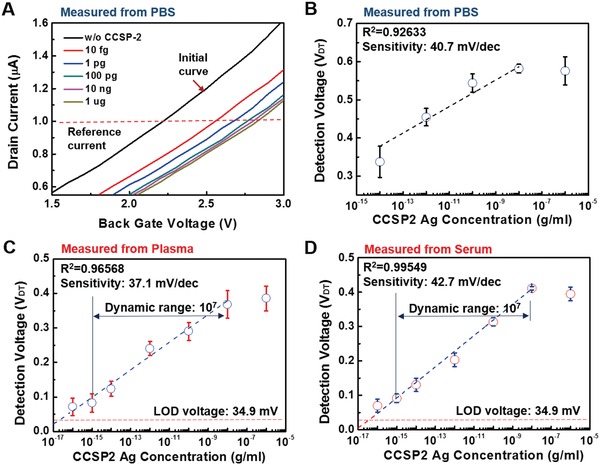
Standard curves of E‐FECS system. A) *I*_D_–*V*_BG_ curves of E‐FECS according to CCSP‐2 concentrations in 1× PBS measured with the modified disposable multiwell gate. Variation of *V*_DT_ according to different CCSP‐2 concentrations was measured in B) 1× PBS, C) plasma, and D) serum. The dynamic range of the E‐FECS system was determined from 10^−15^ to 10^−8^ for quantitative analysis and it showed a good linearity. (1× PBS: *R* ^2^ = 0.92633, Plasma: *R* ^2^ = 0.96568, Serum: *R* ^2^ = 0.99549).

We systematically evaluated the performance of E‐FFES detection for recombinant CCSP‐2 when spiked into serum, plasma, and 1× PBS solutions. The concentration of CCSP‐2 in these samples ranged from 100 ag mL^−1^ to 1 µg mL^−1^. **Figure** **5**A shows the representative drain current (*I*_D_)‐back gate voltage (*V*_BG_) result according to various CCSP‐2 concentrations measured with anti‐CCSP‐2‐immobilized DMWG connected with E‐FECS chip and integrated measurement box. The *I*_D_–*V*_BG_ curves were measured in 1× PBS, plasma, and serum environments and the results were replotted as shown in Figure 5B,C,D respectively. Detection signals were obtained by subtracting the initial signal in 1× PBS from the final signal which reacted in samples for 20 min. The E‐FECS system showed similar sensitivities in all solutions (1× PBS: 40.7 mV dec^−1^, Plasma: 37.1 mV dec^−1^, Serum: 42.7 mV dec^−1^), but it showed more stable and higher linearity in plasma and serum (Plasma *R* ^2^: 0.96568, Serum *R* ^2^: 0.99549) than in 1× PBS (*R* ^2^: 0.92633). In plasma and serum samples, the E‐FECS had a dynamic measurable range of 10^7^ for quantitative analysis of CCSP‐2.

### 2.6 Clinical Validation of CCSP‐2 Detection Using E‐FECS System for CRC Diagnosis

**Figure 6:**
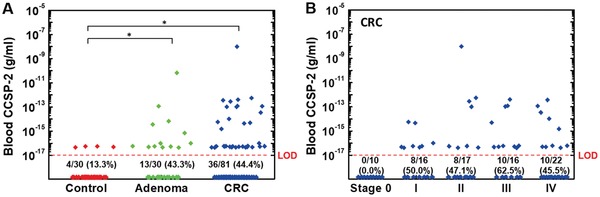
A) Clinical validation of plasma CCSP‐2 detection using an E‐FECS system in controls (*n* = 30, red diamonds) and in patients with colorectal adenoma (*n* = 30, green diamonds) and colorectal cancer (CRC; *n* = 81, blue diamonds). The levels of plasma CCSP‐2 were significantly elevated in samples from CRC and adenoma compared with those from the controls (*P < 0.001). B) Plasma CCSP‐2 measurement from CRC presented by cancer stage (0–IV). The positive result for plasma CCSP‐2 did not differ across Stages I–IV (*P* = 0.603). The red dot line represents the lower limit of detection (LOD) for E‐FECS.

We next compared the detection capacity of the E‐FECS for CCSP‐2 in plasma samples of control subjects versus those with CRC or with colon adenomas. We classified the samples as positive if CCSP‐2 was detected above the LOD (10^−17^ g mL^−1^). CCSP‐2 was detected more frequently in CRC and advanced adenoma plasma samples than in control samples (CRC: 44.4%, adenoma: 43.3%, vs control: 13.3%; *P* = 0.001, **Figure** **6**A). The levels of CCSP‐2 protein were also significantly higher in samples from CRC and advanced adenoma compared with those from control subjects (both *P* < 0.001). There was no significant association between the intensity of the tissue IHC staining and the plasma CCSP‐2 levels (*P* = 0.789). Detection of CCSP‐2 was further analyzed according to the clinical stages of the CRC patients. As shown in Figure 6B, no significant difference in the plasma CCSP‐2 positivity was observed across cancer stages I–IV (*P* = 0.603), and CCSP‐2 was not detected in cancer stage 0. No noticeable differences in other clinicopathologic characteristics were found between CRC cases who were negative for CCSP‐2 in plasma compared with those who were positive (Table S3, Supporting Information). The overall sensitivity and specificity of blood CCSP‐2 in the diagnosis of all colorectal tumors, CRC plus advanced adenoma, were 43.3% and 86.7%, respectively (AUC was 0.70, 95% confidence interval CI 0.61‐0.79). For CRC versus control subjects, sensitivity and specificity were 44.4% and 86.7%, respectively (AUC of 0.67, 95% CI 0.57‐0.77); advanced adenomas showed similar results, at 43.3% sensitivity and 86.7% specificity (AUC of 0.67, 95% CI 0.53‐0.80).

### 2.7 Comparison and Combination of Carcinoembryonic Antigen (CEA) with CCSP‐2

**Figure 7:**
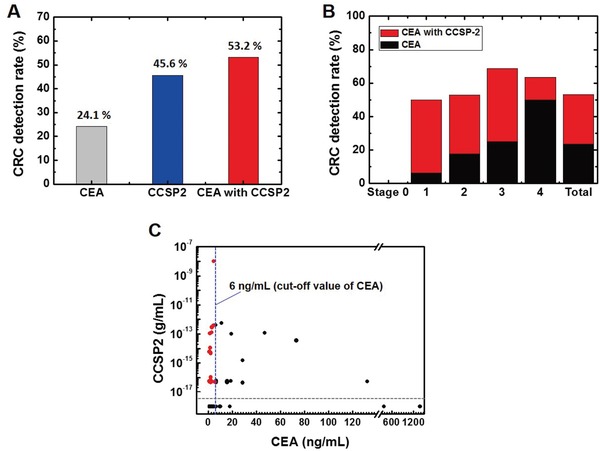
Comparison of CEA with CCSP‐2 in colorectal cancer (CRC) detection. A) CCSP‐2 showed a higher CRC detection rate compared to CEA (24.1% vs 45.6%) and the combination of CCSP‐2 with CEA allowed for detection in a higher number of cases (53.2%). B) CRC detection rate graphed by cancer stage (0–IV). The combination of CCSP‐2 and CEA improved the CRC detection rate, especially for early stage cancer. C) CCSP‐2 level versus CEA level for each CRC patient. The vertical line indicates the cutoff value for CEA, and the horizontal line indicates the lower limit of detection for E‐FECS. The red dots, which are located on the left side of the vertical line and above the horizontal line, represent CRC cases characterized by high CCSP‐2 and low CEA (*n* = 23, 29%).

CEA values were available for 79 out of the 81 CRC patients, except for two cases with intramucosal cancer. Among the 79 CRC patients, only 19 (24.1%) exhibited elevated serum CEA level (**Figure** **7**A,B). By using CCSP‐2, more cases were detected (36 [45.6%]), and the combination of CCSP‐2 with CEA (i.e., positive CCSP‐2 or elevated CEA) was considered a positive result, which allowed for detection of a greater number of cases (42 [53.2%]). The two markers were mostly uncorrelated (kappa value 0.162, *P* = 0.099), with some tumors only characterized by high CEA values and others only by high CCSP‐2 (Figure 7C).

## 3 Discussion

We report the first detection of CCSP‐2 in blood from human CRC patients and show that this circulating protein biomarker is equally sensitive for the detection of early (Stage I–II) tumors as well as overtly metastatic (Stage IV) disease. Plasma CCSP‐2 showed comparable sensitivity for detection of advanced adenomas and of CRCs, providing the first proof of the utility of blood biomarker detection for curable CRC precursor lesions. Furthermore, we demonstrate that a substantial fraction of CRC tumors negative for serum CEA were detected as positive for plasma CCSP‐2, showing that these two markers are relatively uncorrelated. Our results yielded a 86.4% specificity for CCSP‐2 in normal control individuals, which is close to the previously pooled estimate for specificity of 89% for CEA.^24^

Currently, postoperative monitoring of serum CEA for early detection of CRC relapse is the standard of clinical care^25^; accordingly, our findings suggest that a two‐marker panel composed of CEA and CCSP‐2 would provide increased sensitivity for early detection of potentially resettable disease recurrence, particularly for CRC cases that show high expression for CCSP‐2 but not for CEA. The 89% specificity of serum CEA has been considered too low for use as a single marker for general screening for colon cancer; however, serum CEA recently showed promise for colon cancer screening when used as part of a multianalyte test that combined CEA together with mutation detection from circulating tumor DNA, which showed a 99% specificity.^24^, ^25^, ^26^ This multianalyte panel detected ≈63% of stage I–III CRCs, suggesting an opportunity for additional contribution by markers such as CCSP‐2 that show high sensitivity to early stage CRC. Moreover, the high sensitivity of CCSP‐2 for detecting advanced adenomas suggests its potential utility in multianalyte panels for detecting such cancer precursor lesions.

CRC is the second‐leading cause of cancer‐related deaths worldwide, and detection at early clinical stage can reduce cancer‐related mortality.^1^, ^2^, ^27^ Development of a blood‐based assay extends the benefits of colon cancer screening to a substantial portion of individuals who are not willing to undergo current screening methods such as colonoscopy or stool‐based tests.^3^, ^4^, ^10^ As the current sensitivity of 63% of multianalyte blood panels still lags behind that of FIT (pooled sensitivity of 79%),^12^, ^13^, ^14^, ^15^, ^16^, ^17^ development of a new complementary blood marker such as CCSP‐2 remains an important subject for translational research. In addition, although it is difficult for blood‐based assays to completely replace current screening methods, such as FIT, a combination of these two approaches could help to increase sensitivity and specificity, while reducing the number of false positives that occurring using only one method.

We also developed a highly sensitive field effect transistor (FET)‐based biosensor, the E‐FECS, for use as single antibody‐based detection technology for identifying cancer biomarkers in blood. Current biosensors are limited in terms of low stability and sensitivity for liquid‐based samples, and the E‐FECS was developed in order to overcome such issues. In contrast to the bulky nature of previous FET‐based electrochemical biosensors, the E‐FECS biosensor has a compact structure consisting of a small measurement box, a sensor in the shape of a chip, and a DMWG treated with surface stabilization chemistry. Moreover, the structure of E‐FECS has been tailored on nanoscale to efficiently detect minute amounts of target biomarker in blood samples, thereby achieving greater sensitivity than those of commercial FET sensors. In particular, the 1505 mV/pH sensitivity of the E‐FECS represents a 25‐fold improvement over those of commercial sensors. Furthermore, the surface of the DWMG of the E‐FECS is subject to specific treatment that provides stability for analysis of liquid samples and that minimizes interferences by other substances in the blood that often hinder measurements using FET‐based biosensors.^28^, ^29^, ^30^, ^31^ In particular, the sensor used in our current study was capable of reading CCSP‐2 protein in blood to a limit of 100 ag mL^−1^ and provided quantitative analysis across 7 orders of magnitude in the 1 fg mL^−1^ to 10 ng mL^−1^ range.

Our 44% detection rate of CCSP‐2 in the blood of CRC patients is lower than expected, as CCSP‐2 was shown to be increased by more than 8‐fold in > 80% of CRC cases.^18^ Possible explanations for this phenomenon include low level of plasma CCSP‐2 and CCSP‐2 protein degradation. Further directions of technical improvements for detecting CCSP‐2 may include targeting additional CCSP‐2 peptides for E‐FECS detection or employing (Fab) antibody fragments for the E‐FECS system.

One limitation of this study is the lack of comparison between the diagnostic performance of CCSP‐2 as a single marker and the combination of the CEA using ROC curve. This occurred because the serum CEA in the control group was not measured. Further clinical evaluations are warranted to directly compare the performance of CCSP‐2 with CEA or other screening tests in the same cohort in order to assess the predictive abilities of CCSP‐2 and evaluate the benefits of combining it with other methods. Further studies would also help to validate the performance of CCSP‐2 by measuring it against an independent cohort.

## 4 Conclusion

Our study reports the first detection of CCSP‐2 in blood from human CRC patients by using newly developed E‐FECS system. Our study demonstrates the clear potential of CCSP‐2 as a blood‐based biomarker for CRC, with a wide range of detection that includes early stage cancers and advanced adenoma. Furthermore, a two‐marker panel combining CEA and CCSP‐2 significantly increased the detection rate for CRC. Therefore, the use of CCSP‐2 in combination with the E‐FECS system may contribute to the development of improved blood‐based marker panels for postoperative monitoring and early detection of CRC.

## 5 Experimental Methods

*Fabrication of the E‐FECS System*: The E‐FECS was fabricated using a 6‐inch p‐type (100) silicon‐on‐insulator wafer that had a 750 nm thick buried oxide layer and a 100 nm thick top silicon layer. The active region of the E‐FECS was formed via a combination of photolithography and inductively coupled plasma reactive‐ion etching (ICP‐RIE) processes. The 10 nm thick top gate oxide layer was formed using a dry oxidation process. Arsenic ions were implanted into the source and drain regions (concentration: 3 × 10^15^). A Ti/TiN/Al/TiN multilayer was deposited on the gate oxide layer using a sputtering system and etched by ICP‐RIE to form the gate electrode structure. To form the contact pads on the source and drain regions, the Ti/TiN/Al/TiN multilayer was deposited using the sputtering system.^20^ The final E‐FECS fabricated on 6‐inch wafer was diced into many pieces, each containing an E‐FECS.^32^ Each of these pieces was packaged with an epoxy layer and integrated with a printed circuited board. In order to replace a heavy probe station, a small electric‐circuit integrated measurement box was also developed (Figure 1A).

To fabricate the DMWG, a thin SnO_2_ (99.9%) film was deposited on the top of an indium tin oxide (ITO) glass substrate (ITO thickness: 300 nm). The polydimethylsiloxane (PDMS) well was prepared using a Sylgard 184 Silicon Elastomer Kit (Dow Corning, Seoul, Republic of Korea) including a base and a curing material. To form the PDMS well, the base and a curing material were mixed at 10:1 ratio, and baked at 60 °C overnight after bubble removal with a vacuum pump. To combine the PDMS with the ITO glass with SnO_2_, both were initially sonicated for 1 min in deionized water and ethanol, respectively. They were then dried using N_2_ gas, and were subjected to O_2_ plasma with 70 W of power and 30 sccm of O_2_ gas flow for 1 min. Then, the PDMS and ITO glass were attached to form the DMWG by employing an annealing process at 60 °C for 10 min.^21^, ^33^

*Surface Modification of the DMWG*: The surface of the DMWG was treated with O_2_ plasma system at 70 W for 1 min to form OH groups, and 5% 3‐aminopropyltriethoxysilane (APTES) was treated for 1 h to form NH_2_ groups. The DMWG was then washed with ethanol and baked at 120 °C for 30 min. 2.5% glutaraldehyde was prepared in 2‐[4‐(2‐hydroxyethyl)piperrazin‐1‐yl]ethanesulfonic acid (HEPES) and added on the DMWG surface for 1 h to create amide bonds with antibodies. After glutaraldehyde was washed with HEPES, the DMWG was incubated with 5 µg mL^−1^ CCSP‐2 antibodies diluted in PBS for 1 h at room temperature. After washing with PBS, 1 m ethanolamine was added for 1 h to remove unreacted COOH groups. Then, the DMWG was incubated with 5% bovine serum albumin (BSA) for 1 h to block nonspecific adsorption from blood biomolecules (Figure 2A).

*Measurement Setup and Stability Test of the E‐FECS*: A commercial Leak‐Free reference electrode (Harvard Apparatus, 69‐0023) and a dual channel system source meter (Keithley, 2634B) were utilized for the E‐FECS measurement. The drain‐source current was measured against a sweep in the bottom gate voltage (−10 to +10 V) at a fixed drain voltage of 6 V. The reference electrode in the solution was electrically grounded and the DMWG was electrically connected to the top gate of the E‐FECS. The electrical stability of the DMWG was evaluated in blood and 1× PBS environments. Voltage signals generated by reaction of 1× PBS and blood with selected three wells were measured, and initial and final voltage signal levels of each solution were compared (Figure 2B,C,D).

*Study Population*: Fresh‐frozen colorectal tumor tissues, paired normal colorectal tissues, and plasma specimens from patients with colorectal tumors (10 advanced adenomas greater than 1 cm in size and 77 cancers) were provided by Asan Bio‐Resource Center (BRC), Korea Biobank Network (2016‐9[121]). These tumor samples were excised from patients during surgery, and the plasma specimens were obtained before surgery. The plasma and tumor samples were randomly divided into two groups for E‐FECS verification assay (seven cases of stage II or III cancer, Table 1A) and clinical validation (80 cases: 10 advanced adenomas and 70 cancers, Table 1B). Additionally, 31 plasma specimens were collected from 31 patients with colorectal tumors (20 advanced adenomas greater than 1 cm in size and 11 cancers) who were prospectively enrolled from the outpatient clinic of the Department of Gastroenterology at Asan Medical Center. These 31 blood samples were also included in the clinical validation set. The clinicopathologic characteristics of the patients in the colorectal tumor groups are shown in Table 1B.

Control subjects with no colorectal adenomas or cancer detected by complete colonoscopies were also prospectively enrolled. Exclusion criteria for control subjects included the following: any malignant disease; a family history of familial polyposis or Lynch syndrome; any colorectal disease such as inflammatory bowel disease. A total of 37 control subjects were enrolled, and the samples were randomly divided into two groups for biosensor verification assay (*n* = 7) and clinical validation (*n* = 30), as shown in Table 1A,B. The study protocol was approved by the institutional review board of Asan Medical Center, and informed consent was obtained from all subjects (protocol no. 2015‐0969).

*Blood Collection and Processing*: BRC plasma samples were collected in sodium citrate‐containing tubes before surgery, and processed at the Asan BRC 1 h after collection. About 5 mL of blood was centrifuged at 1900 × g at 4 °C for 10 min, and the supernatant was centrifuged again at 1600 × g at 4 °C for 10 min to separate plasma from the whole blood. The resulting supernatants were stored at −196 °C until use. Prospectively collected plasma samples were collected in sodium citrate‐containing tubes, and processed 1 h after collection. About 5 mL of blood was centrifuged at 1900 × g at 4 °C for 20 min to separate plasma from whole blood. The resulting supernatants were stored at −80 °C until use.

*Tissue Microarray Construction*: For CCSP‐2 expression analysis, three tissue microarray slides were established using formalin‐fixed paraffin‐embedded tissues from 79 colorectal neoplasms and paired normal colorectal tissues. Representative areas of the tumors were marked on hematoxylin and eosin‐stained slides. Duplicates of three cores from the tumor tissues and one core from the paired normal colorectal tissue measuring 1.5 mm in diameter were arrayed from the corresponding paraffin blocks into a recipient block using an arraying machine (Tissue microarrayer; Pathology Devices, Westminster, MD, USA). The first 4 µm sections from these arrays were examined for validation purposes, and subsequent sections were used for IHC.

*IHC*: Immunohistochemical staining of CCSP‐2 was performed using tissue microarray slides with the BenchMark XT automatic immunostaining device (Ventana Medical Systems, Tucson, AZ, USA) according to the manufacturer's instructions. Detailed immunohistochemical staining procedure was described in the previous study.^19^ In brief, 4 µm tissue sections were transferred onto silanized charged slides, dried for 10 min at room temperature, and incubated for 20 min at 65 °C. Antigens were retrieved by heating the sections for 64 min in Cell Conditioning 1 (CC1) buffer. After anti‐CCSP‐2 antibody (1:100) incubation for 32 min in the autoimmunostainer, detection was carried out with the Ventana OptiView DAB IHC Detection Kit (Ventana Medical Systems). The expression profiles of CCSP‐2 were scored according to staining intensity (0, negative; 1+, weak positive; 2+, strong positive), because the staining for CCSP‐2 was homogenous in the stained core.

*ELISA*: The wells of microtiter plates were coated (O/N, 4 °C) with 5 µg mL^−1^ of the CCSP‐2 monoclonal antibody in 100 µL of a coating buffer (0.05 m Na_2_CO_3_, 0.05 m NaHCO3), and blocked with 2% BSA in Tris‐buffered saline with Tween 20 (TBST) for 1 h at 37 °C. Then, 100 µL of the samples were loaded in triplicates and incubated for 1 h at room temperature, followed by addition of 100 µL of anti‐CCSP‐2 antibody 1 (1:1000, Cloud‐Clone Corp.) for an additional 1 h at room temperature. Horseradish peroxidase (HRP)‐conjugated goat anti‐rabbit immunoglobulin G (IgG) (1:5000 in blocking buffer) was added (1 h, room temperature), followed by addition of 50 µL of chromogenic substrate (TMB) for 30 min. The substrate reaction was stopped with 100 µL of 2 m H_2_SO_4_, and the absorbance at 450 nm was measured using an ELISA plate reader. Plates were washed five times with washing buffer (TBS, pH 7.4, containing 0.1% [v/v] Tween 20) after each step. As a reference for quantification, a standard curve was established by serial dilution of recombinant CCSP‐2 protein (10 pg mL^−1^ ≈ 0.3 µg mL^−1^).

*Detection of CCSP‐2 in Blood with the E‐FECS System*: Electrical signal generated by CCSP‐2 antigen‐antibody reaction in E‐FECS system was measured using Keithley 2634B. For quantitative analysis of the detected CCSP‐2, a standard curve was first established through the measurement of serially diluted recombinant CCSP‐2 protein with 1× PBS, untreated serum, and untreated plasma (100 ag mL^−1^, 1 µg mL^−1^). The initial voltage signal was obtained by measuring *I*_D_–*V*_BG_ curves in 1× PBS. The solution was then removed and the plasma specimens were directly injected into the anti‐CCSP‐2‐immobilized DMWG and allowed to react for 20 min. After washing with a 1× PBS solution, the washed sample was again removed from the well. Finally, a clean 1× PBS solution was injected again and the final voltage signal was measured. The detection voltage signal measurement was calculated by subtracting the final (20 min) voltage signal and the initial (0 min) voltage signal at the reference current. The detection signal was then compared with the standard curve for calculation of CCSP‐2 concentration.

*Statistical Analysis*: Differences in continuous variables between two groups were evaluated using the Student's t‐test or the Mann–Whitney U test, and differences in categorical variables were evaluated with the *X* ^2^ test or Fisher's exact test. Associations between two continuous variables were evaluated using bivariate correlation analysis. The areas under receiver operating characteristic (ROC) curves (AUCs) provided a measure of the overall performance of the diagnostic test. Statistical significance was defined as a *P* value of < 0.05, and the SPSS statistical software (version 20.0; SPSS, Chicago, IL) was used for all analyses.

## Conflict of Interest

The authors declare no conflict of interest.

## Supporting information

Supplementary
