# Supplementary material for: A Novel Blood‐Based Colorectal Cancer Diagnostic Technology Using Electrical Detection of Colon Cancer Secreted Protein‐2
Source: Adv Sci (Weinh). 2019 Apr 16;6(11):1802115. doi: 10.1002/advs.201802115 (PMC6548955; doi:10.1002/advs.201802115)
Supplement: Supplementary file 1 — Supplementary [file ADVS-6-1802115-s001.pdf]

Copyright WILEY-VCH Verlag GmbH & Co. KGaA, 69469 Weinheim, Germany, 2019.

Supporting Information

for *Adv. Sci.,* DOI: 10.1002/advs.201802115

A Novel Blood-Based Colorectal Cancer Diagnostic Technology Using Electrical Detection of Colon Cancer Secreted Protein-2

Minhong Jeun, Hyo Jeong Lee, Sungwook Park, Eun-ju Do, Jaewon Choi, You-Na Sung, Seung-Mo Hong, Sang-Yeob Kim, Dong-Hee Kim, Ja Young Kang, Hye-Nam Son, Jinmyoung Joo, Eun Mi Song, Sung Wook Hwang, Sang Hyoung Park, Dong-Hoon Yang, Byong Duk Ye, Jeong-Sik Byeon, Jaewon Choe, Suk-Kyun Yang, Helen Moinova, Sanford D. Markowitz, Kwan Hyi Lee, and Seung-Jae Myung**

Copyright WILEY-VCH Verlag GmbH & Co. KGaA, 69469 Weinheim, Germany, 2019.

Supporting Information

A Novel Blood-Based Colorectal Cancer Diagnostic Technology Using Electrical Detection of Colon Cancer Secreted Protein-2

Minhong Jeun, Hyo Jeong Lee, Sungwook Park, Eun-ju Do, Jaewon Choi, You-Na Sung, Seung-Mo Hong, Sang-Yeob Kim, Dong-Hee Kim, Ja Young Kang, Hye-Nam Son, Jinmyoung Joo, Eun Mi Song, Sung Wook Hwang, Sang Hyoung Park, Dong-Hoon Yang, Byong Duk Ye, Jeong-Sik Byeon, Jaewon Choe, Suk-Kyun Yang, Helen Moinova, Sanford D. Markowitz, and Kwan Hyi Lee and Seung-Jae Myung**

Table 1. Stability of the electrical detection signals (bare DMWGs and modified DMWGs)

**Bare DMWGs**	**1** (ΔmV)	**2** (ΔmV)	**3** (ΔmV)	**SD** (ΔmV)	**CV (%)**
Initial PBS	0	0	0		
Blood 0 min	193.57	206.29	268.06	39.85	17.90
Blood 5 min	127.34	186.58	229.32	51.21	28.28
Blood 10 min	82.06	165.43	206.88	63.57	41.97
Blood 15 min	45.53	148.44	183.72	71.80	57.03
Blood 20 min	23.12	136.49	167.1	75.85	69.65

**Modified DMWGs**	**1** (ΔmV)	**2** (ΔmV)	**3** (ΔmV)	**SD** (ΔmV)	**CV (%)**
Initial PBS	0	0	0		
Blood 0 min	269.31	285.29	306.78	18.80	6.55
Blood 10 min	433.32	443.1	443.6	5.80	1.32
Blood 20 min	495.54	498.57	492.6	2.99	0.60
PBS 0 min	39.49	34.17	31.06	4.26	12.21
PBS 10 min	49.94	44.57	38.87	5.54	12.45

DMWG, disposable multi-well gate

Table 2. Comparison of the clinicopathologic characteristics between CCSP-2-weak-positive and strong-positive colorectal cancer patient groups

**Variables**	**Weak (n = 39)**	**Strong (n = 30)**	***P***
Age, years, median (range)	58 (37–80)	64 (30–78)	0.877
Sex, male, no. (%)	21 (53.8%)	16 (53.3%)	0.966
Location, no. (%)[a]			0.751
Proximal	18 (46.2%)	15 (50.0%)	
Distal	21 (53.8%)	15 (15.0%)	
Stage, no. (%)			0.111
0	3 (7.7%)	2 (6.7%)	
I	5 (12.8%)	10 (33.3%)	
II	9 (23.1%)	6 (20.0%)	
III	8 (20.5%)	6 (20.0%)	
IV	14 (35.9%)	6 (20.0%)	
Differentiation, no. (%)			0.003
Well	2 (5.1%)	8 (26.7%)	
Moderate	33 (84.6%)	22 (73.3%)	
Poor	4 (10.3%)	0 (0.0%)	
CEA, median (range), ng/mL	1.8 (0.3–1380)	2.1 (0.5–132.0)	0.443

[a] Proximal colon includes cecum, ascending colon, and transverse colon; Distal colon includes descending colon, sigmoid colon, and rectum.

Table 3. Comparison of the clinicopathologic characteristics between blood CCSP-2-negative and positive colorectal cancer patient groups

**Variables**	**Negative (n = 45)**	**Positive (n = 36)**	***P***
Age, years, median (range)	61 (30–78)	59 (36–80)	0.949
Sex, male, no. (%)	22 (48.9%)	22 (61.1%)	0.273
Location, no. (%)[a)]			0.177
Proximal	23 (51.1%)	13 (36.1%)	
Distal	22 (48.9%)	23 (63.9%)	
Stage, no. (%)			0.067
0	10 (22.2%)	0 (0.0%)	
I	8 (17.8%)	8 (22.2%)	
II	9 (20.0%)	8 (22.2%)	
III	6 (13.3%)	10 (27.8%)	
IV	12 (26.7%)	10 (27.8%)	
Differentiation, no. (%)			0.232
Well	11 (24.4%)	3 (8.3%)	
Moderate	31 (68.9%)	32 (88.9%)	
Poor	3 (6.7%)	1 (2.8%)	
CEA, median (range), ng/mL	1.5 (0.3–1380.0)	2.9 (0.8–132.0)	0.397

[a)] Proximal colon includes cecum, ascending colon, and transverse colon; Distal colon includes descending colon, sigmoid colon, and rectum.
